# 779 Burn Therapists’ Perspective of Rehabilitation and Recovery Post Primary Closure of a Burn Wound

**DOI:** 10.1093/jbcr/irae036.320

**Published:** 2024-04-17

**Authors:** Breanna Potter, Heather S Dodd, Britni Korshin

**Affiliations:** UNC Health - NC Jaycee Burn Center, Chapel Hill, NC; UNC Health-NC Jaycee Burn Center, Durham, NC; UNC Health - NC Jaycee Burn Center, Chapel Hill, NC; UNC Health-NC Jaycee Burn Center, Durham, NC; UNC Health - NC Jaycee Burn Center, Chapel Hill, NC; UNC Health-NC Jaycee Burn Center, Durham, NC

## Abstract

**Introduction:**

Primary closures have been used as a surgical intervention to close burn wounds within a timely fashion to decrease risk for infection, minimize scarring, and optimize wound healing. However, when a primary closure is performed on or near a body’s joint, the rate of return to normal function and range of motion (ROM) may be prolonged. Subjective observations include increased pain related to skin tension, increased anxiety, and potential for dehiscence of the primary closure. The purpose of this research is to determine the protocols and perceptions of burn therapists at verified ABA burn centers in regard to their patient’s rate of return to normal ROM and overall function in ADLs. The results of this study may guide the medical team in providing best practice with regard to primary closure of a burn wound.

**Methods:**

An electronic survey was administered to deem understanding of burn therapists’ perception of challenges and/or advantages of ROM post-primary closure near a joint of the body. The inclusion criteria for those being surveyed include: a) Licensed Burn Therapists (OT/PT) within ABA verified burn centers, b) Literate in English language.

**Results:**

Up to 83% of therapists surveyed reported that primary closures placed over a joint have been used as a surgical intervention at their facility; the data indicated that burn therapists are likely to provide intervention to this patient population. From our research, we found that 65% of burn therapists report there is not a standard ROM protocol following a primary closure over a joint. AROM is most initiated POD 1 and PROM is most commonly initiated POD 3 – 5. The most frequently reported considerations when initiating ROM include: stretching within a patient’s pain tolerance and the ability to visualize tension to the wound. Majority of burn therapists report their patients fully regain ROM to the affected joint, as well as their baseline independence with ADLs after a primary closure, in which 10% of burn therapists reported these were always achieved.

**Conclusions:**

A primary closure is defined as healing by primary intention in clean wounds with minimal tissue loss that are amenable to approximation of wound edges. Our therapy team has observed both advantages and challenges when these closures have been placed over a bodily joint. Results encourage developing therapy protocols to allow for coordination of therapy with wound care to prevent complications, allow for proper pain management strategies, and provide an opportunity for therapists to visualize the wound during therapeutic activities and ROM.

**Applicability of Research to Practice:**

The results of this study will aim to guide the medical team in developing a therapy protocol for best practice when initiating therapy after a primary closure. Ultimately, direct ongoing collaboration with our surgeons to determine best practice during surgical interventions will optimize patients' functional outcomes, with decreased risk for wound dehiscence and increased pain.

